# Breast cancer screening awareness, practice, and perceived barriers: A community‐based cross‐sectional study among women in south‐eastern Bangladesh

**DOI:** 10.1002/hsr2.1799

**Published:** 2024-01-10

**Authors:** Mohammad Injamul Hoq, Shamima Jahan, Md. Hasan Mahmud, Md. Mayin Uddin Hasan, Md. Jakaria

**Affiliations:** ^1^ School of Public Health, Epidemiology and Social Medicine at the Institute of Medicine, Sahlgrenska Academy University of Gothenburg Gothenburg Sweden; ^2^ Department of Public Health University of Creative Technology Chittagong Chittagong Bangladesh; ^3^ Department of Pharmaceutical Sciences State University of Bangladesh Dhaka Bangladesh; ^4^ Department of Pharmacy International Islamic University Chittagong Kumira Chittagong Bangladesh; ^5^ The Florey Institute The University of Melbourne Parkville Victoria Australia

**Keywords:** breast cancer, breast screening, BSE, CBE, perceived barrier

## Abstract

**Background and Aims:**

Breast cancer is a leading cause of mortality in Bangladesh. An early‐stage screening is the best way to reduce both the morbidity and mortality burden of breast cancer. The study evaluated awareness, practice, and perceived barriers toward breast cancer screening in Bangladesh.

**Methods:**

A community‐based cross‐sectional study was conducted from October 2021 to December 2022 in Chattogram, Bangladesh, where 869 women (18 years or above) were randomly selected in this study.

**Results:**

Among 869 participants, 47.3% of women were recruited from urban areas and 52.7% participated from rural areas. Only 32.68% of respondents (urban vs. rural: 44.28% vs. 22.27%) were aware of breast self‐examination (BSE) and 52.47% of respondents (urban vs. rural: 63.75% vs. 42.36%) had ever heard Clinical Breast Examination (CBE), respectively. Among the respondents, 27.73% (urban vs. rural: 40.15% vs. 16.59%) performed their BSE, and only 14.61% of respondents (urban vs. rural: 21.90% vs. 8.08%) had ever visited for CBE. Women residing in rural areas were approximately three times (AOR: 0.36 [95% CI: 0.25–0.52], AOR: 0.37 [95% CI: 0.23–0.58]) less likely to perform BSE and CBE, respectively, than urban dwellers. We found that higher‐educated women tend to do more BSE and CBE than women with low levels of education. Perceptions of having “no symptoms” and being “risk‐free” are leading barriers to breast screening among women.

**Conclusion:**

Poor awareness and practice were observed in screening among the urban and rural women in Bangladesh. Urban area dwellers had comparatively better understanding and practice than rural dwellers. We think extending health education and health promotion activities toward breast cancer screening is essential in this region.

## 1 BACKGROUND

Breast cancer is a heterogeneous disease that is increasingly prevalent in developed and developing countries and is one of the most commonly occurring cancers worldwide. Over two million breast cancer cases were detected worldwide, with 6,27,000 deaths in 2018.^1^ The incidence and mortality rates of breast cancer are varied in developing countries and developed countries. The highest incidence of breast cancer has been observed in developed regions, including Australia, Europe, and America, but the mortality rate is relatively higher in developing countries from Africa, Asia, and the Caribbean.^2^ The low incidence rate of breast cancer in developing countries is probably due to a lack of population registry data on breast cancer incidence and recognition of the disease at the advanced stage rather than at the primary stage.^3^, ^4^

Bangladesh is on the list of developing countries with such inconsistency; according to the GLOBOCAN report in 2020, breast cancer is the leading cause of death (6.2% of all cancers) and the most occurring cancer (19% among all cancers) among women in Bangladesh.^5^ Prioritizing prevention strategies on screening and early detection could reduce the mortality of this deadliest cancer. The Bangladesh Ministry of Health and Welfare established numerous screening facility centers, including at the upazila level, and awareness campaigns have been conducted to make women aware of breast cancer.^6^, ^7^ The number of clinical breast examinations (CBE) has increased over the years, and more positive cases have been detected in these years, which is alarming. In 2017, the number of screened participants was just above 260,000 nationwide, indicating that many cases remained undetected due to a lack of screening.^8^

Poor knowledge and awareness are key factors in not practising breast self‐examination (BSE).^9^ However, screening practice and its associated factors among Bangladeshi women are yet to be established by epidemiological studies. A few studies reported poor knowledge and awareness of breast cancer among Bangladeshi women^10^, ^11^, ^12^, ^13^, ^14^ However, these studies were on either hospital‐based or specified group. Little evidence is available on breast cancer screening awareness and its perceived barrier by the women of this region with poor awareness on breast cancer who seek treatment at the later stage of breast cancer. A study showed that the mortality rate of breast cancer can be reduced by 25%–30% with the application of appropriate treatment and early detection of breast cancer by BSE, CBE, and mammography.^15^ This community‐based study aims to assess the awareness, practice, and perceived barriers toward breast cancer screening among Bangladeshi women. We also assess the associated factors of breast cancer screening practice among urban and rural women of Bangladesh.

## 2 METHODS

### 2.1 Study design and area

This was a cross‐sectional study of women in the Chattogram district of Bangladesh for the period of 15 months (October 2021 to December 2022). Two thanas (small administrative area in urban area) (Bandar, Halishahar) from urban area and two upazilla (small administrative area in rural area) (Sitakunda, Hathazari) were selected randomly from 16 thanas and 15 upazillas of Chattogram, respectively.

### 2.2 Sampling technique

Multistage random sampling was used to select participants. On the first stage, two thanas from urban area and two upazilas from rural area of Chattogram were randomly selected by using lottery. Then households of thanas and upazilas were selected by utilizing the head and tail of coin procedure. In the last stage, one woman was selected randomly for interview if the household had one or two women and two women were interviewed if the household had more than two women.

### 2.3 Sample size calculation

Study sample was calculated by using the following formula *n* = *Z* ^2^ *pq*/*d* ^2^, where *n* is the required sample size, *p* the expected (0.5) proportion of study population with awareness and practice of breast cancer screening, *q* = 1 − *p*, and is the level of precision at 5%. *n* = 1.96^2^ × 0.5 × 0.5/(0.05)^2^ = 384. By considering a design effect of 1.5 due to multistage sampling and a 20% nonresponse rate, we estimated a total sample size of 691. Rounding up a total of 1000 participants (500 women from urban areas and the same number from rural areas) were approached for the study participation and 869 (86.9%) agreed to participate.

### 2.4 Data collection tools

A structured questionnaire was designed by using previously published studies^11^, ^12^, ^16^, ^17^, ^18^ that adapted validated questions. It consists of four sections, including sociodemographic characteristics, breast cancer examination awareness, practice, and perceived barrier of breast cancer examination. The questionnaire was prepared in English and then translated into local language for better understanding. A face‐to‐face interview was done for each participant by trained female data collectors.

### 2.5 Variables used

#### 2.5.1 The content of the questionnaire was as follows

- *Sociodemographic data*: age (we were looking for participants aged 18 years or more. According to World Health Organization (WHO) women aged 20–39 years should screen their breasts once every two‐3 years by trained healthcare provider and from 40 years once in a year.^19^ Based on the category, we classified age gropus into 18–39 years, 40–49 years, and 50 or more years), marital status (it is divided into two categories: single and married), educational status (it is categorized from primary to postgraduate level education), occupational status (it is classified into student, housewife, and working women: private/public employee), family income (at the national level, the average monthly household income was 32,422 Bangladeshi Taka (BDT), 26,163 BDT in rural, and 45,757 BDT in urban areas according to the household income and expenditure survey 2022.^20^ Based on that, we categorized the family income into less than 20,000 BDT, 20,000–40,000 BDT, 40,001–60,000 BDT and more than 60,000BDT), family type (nuclear and joint/extended), and religion (hindu and muslim).
- General awareness of breast cancer and its screening (awareness was defined as heard about breast cancer, breast cancer screening, BSE and CBE awareness for breast screening).
- BSE and CBE practice for breast cancer screening.
- Frequency of breast cancer screening (BSE and CBE). It is categorized into weekly, monthly and yearly screening.
- Perceived barriers toward breast cancer screening (fear of clinical examination, shy to uncover breasts, social stigma, lack of awareness program, lack of organization working on breast cancer screening, having no sign and symptoms of breast cancer and perceived free from risk of developing breast cancer).

### 2.6 Data analysis

Data analysis was carried out by using STATA statistical package version SE 17.0. Descriptive statistics were used to describe the sociodemographic characteristics of urban and rural area. Univariable and multivariable logistic regression were applied to identify significant predictors of BSE and CBE practice. To analyze the degree to which dependent and independent variables are associated, an odds ratio (OR) and 95% confidence interval (CI) were used. Moreover, perceived barrier toward screening among the women was displayed descriptively.

### 2.7 Ethics issues

The study was approved by the Institutional Review Board (IRB) of the University of Creative Technology Chittagong, Bangladesh Ref: IRB/UCTC/2021/04. Confidentiality of participants data was maintained, and the participants were included in the study with verbal consent.

## 3 RESULTS

### 3.1 Sociodemographic characteristics of participants

Of the 869 respondents, 411 (47.3%) were from urban areas and 458 (52.7%) resided in rural areas. There was no significant difference in the age groups of the participants between the two regions, and most of the participants in urban and rural areas (69.34% and 73.80%) belonged to 18–39 years age group. Besides that, no significant difference was observed in religion types and muslim was the prevalent group in both regions (94.4% and 93.01%). However, types of family, family income, marital status, educational status, and occupational status of the participants were significantly different from the urban to rural regions. Majority of the participants were housewives (80.29% and 87.12%), married (85.40% and 89.96%), from nuclear families (88.32% and 60.48%), completed secondary education (33.82% and 47.82%), having 20,000 to 40,000 BDT family income (55.96% and 57.86%). However, the proportion of higher education and higher income groups is comparatively high in urban areas than in rural areas. The sociodemographic characteristics of urban and rural area participants are tabulated in Table 1.

**Table 1:** Sociodemographic characteristics of study participants.

Variables	Area of living			
	Urban 411– (47.30%)	Rural 458– (52.70%)	Chi‐square	*p* Value
Age group (years)				0.181
18–39	285 (69.34)	338 (73.80)	3.4197	
40–49	69 (16.79)	57 (12.45)		
50 & more years	57 (13.87)	63 (13.76)		
Total	411 (100%)	458 (100%)		
Marital status				**0.041** *
Single	60 (14.60%)	46 (10.04%)	4.1961	
Married	351 (85.40%)	412 (89.96%)		
Total	411 (100%)	458 (100%)		
Types of family			86.5119	**<0.001** ***
Nuclear	363 (88.32%)	277 (60.48%)		
Joint/extended	48 (11.68%)	181 (39.52%)		
Total	411 (100%)	458 (100%)		
Education status				**<0.001** ***
Primary	108 (26.28%)	131 (28.60%)	38.52	
Secondary	139 (33.82%)	219 (47.82%)		
Higher Secondary	67 (16.30%)	65 (14.19%)		
Undergraduate/postgraduate	97 (23.60%)	43 (9.39%)		
Total	411(100%)	458 (100%)		
Religion			0.7067	0.401
Muslim	388 (94.40%)	426 (93.01%)		
Hindu	23 (5.60%)	32 (6.99%)		
Total	411 (100%)	458 (100%)		
Family income				**<0.001** ***
<20,000BDT	100 (24.33%)	142 (31.00%)	14.145	
20,000‐40,000BDT	230 (55.96%)	265 (57.86%)		
40,001‐60,000BDT	49 (11.92%)	32 (6.99%)		
More than 60,000BDT	32 (7.79%)	19 (4.15%)		
Total	411 (100%)	458 (100%)		
Occupational status			10.7992	<**0.001** ***
Student	65 (15.82%)	54 (11.79%)		
Housewife	330 (80.29%)	399 (87.12%)		
Private/public employee	16 (3.89%)	5(1.09%)		
Total	411 (100%)	458 (100%)		

*Note*: Bold values indicate statistically significant at *p* < 0.05.

* *p* < 0.05

*** *p* < 0.001.

### 3.2 Awareness and practice of breast cancer screening

**Figure 1:**
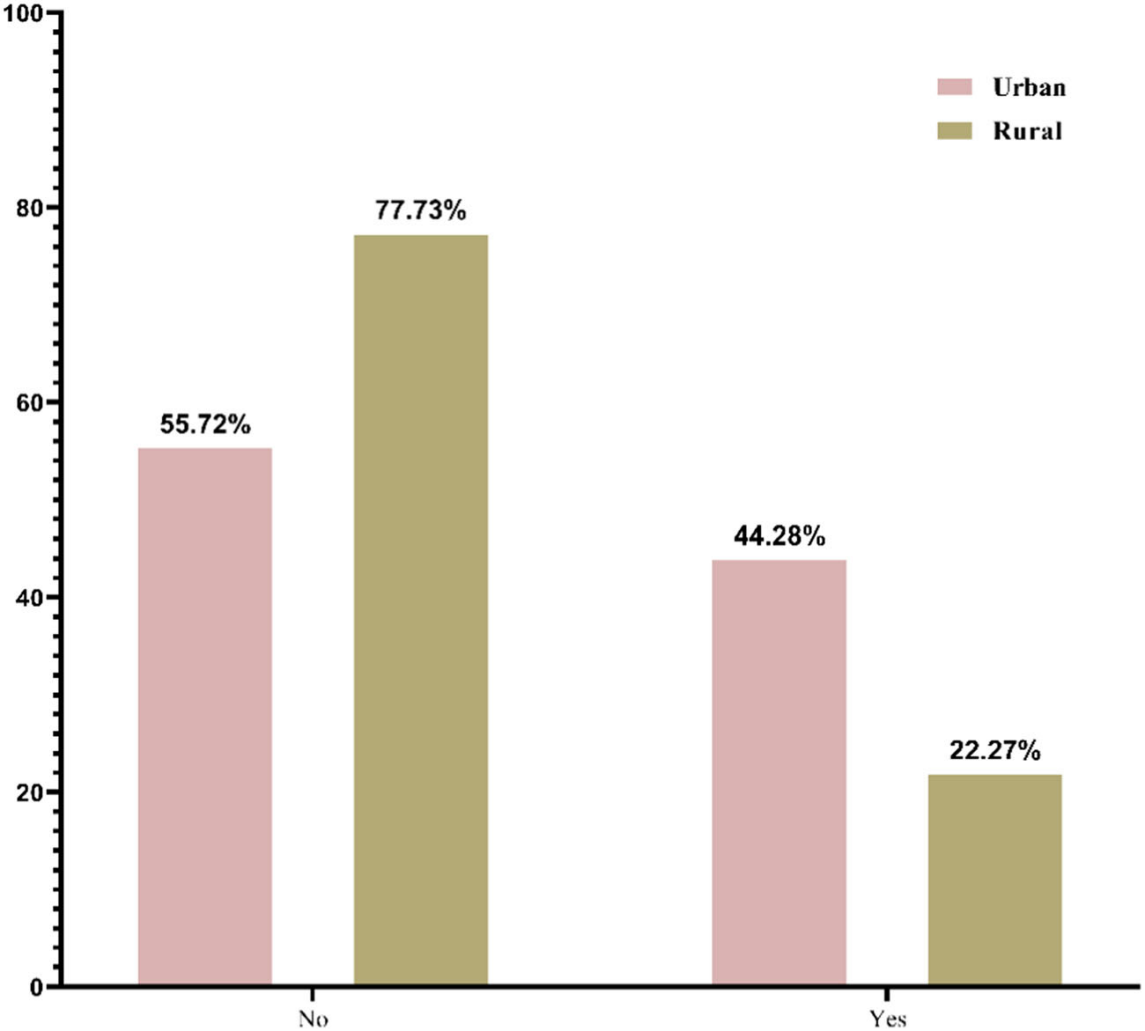
Awareness of breast self‐examination (BSE) among urban and rural women.

**Figure 2:**
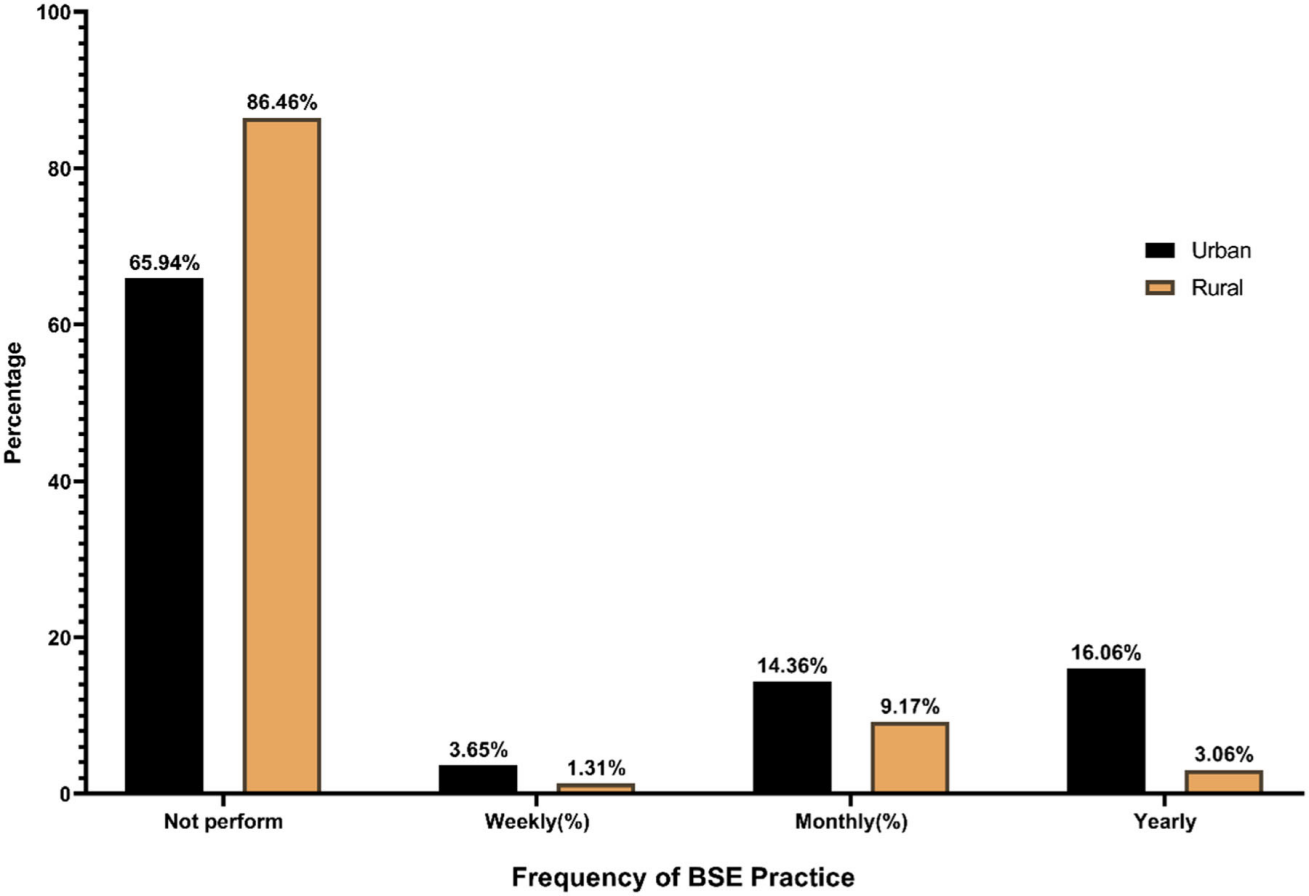
Frequency of breast self‐examination (BSE) practice among urban and rural women.

**Figure 3:**
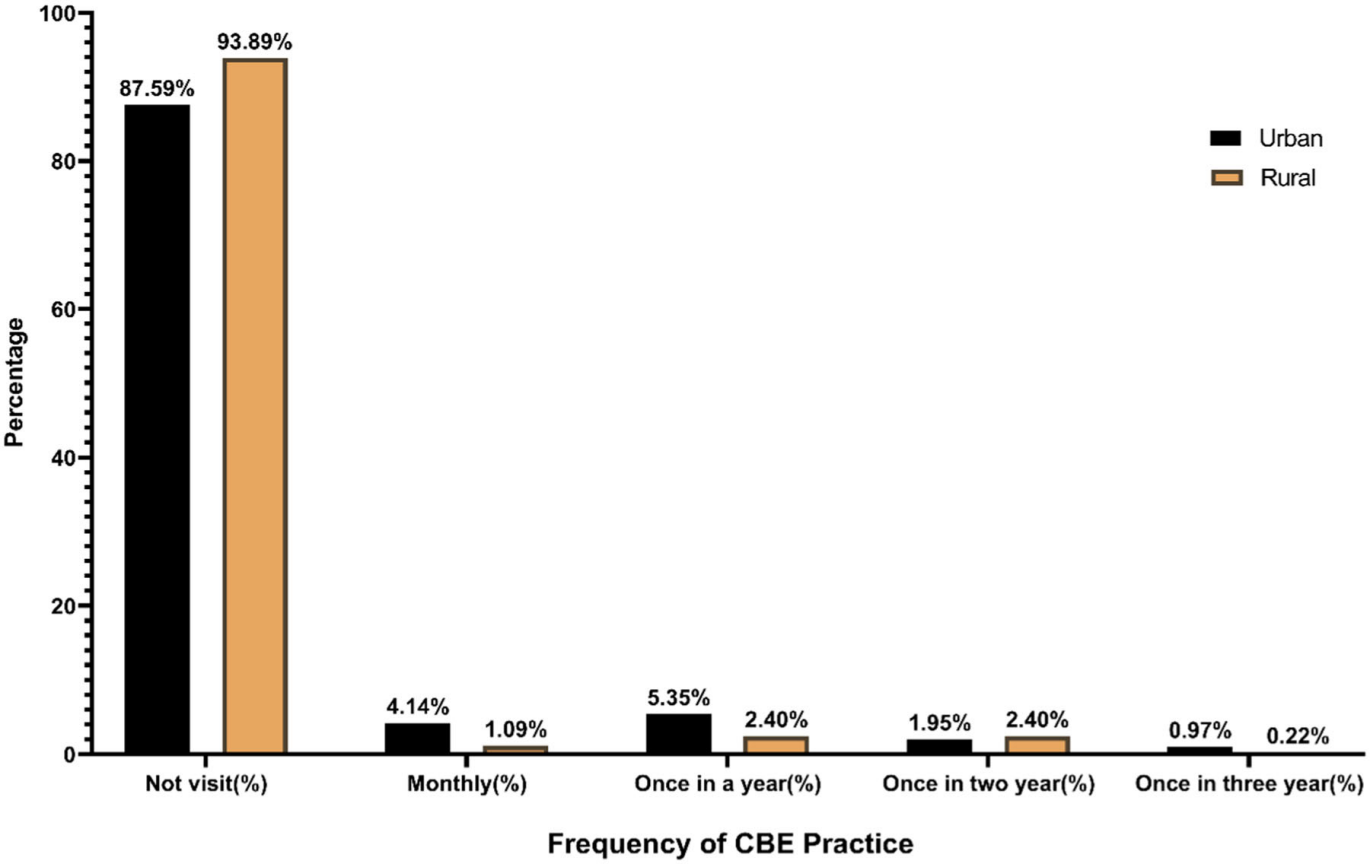
Frequency of clinical breast examination (CBE) practice among urban and rural women.

The proportion was comparatively higher for urban women than rural women in both awareness and practice. About 77% of the respondents (urban vs. rural: 78.10% vs. 75.98%) had heard about breast cancer. Of the total respondents, a vast majority 71.58% (urban vs. rural: 76.16% vs. 67.47%) and 80.20% (urban vs. rural: 81.27% vs. 79.26%) were aware that breast cancer is a preventable and severe disease respectively. However, the finding showed that only 32.68% of respondents (urban vs. rural: 44.28% vs. 22.27%) were aware about BSE (Figure 1) and 52.47% of respondents (urban vs. rural: 63.75% vs. 42.36%) had ever heard CBE. In contrast, a total of 27.73% of respondents (urban vs. rural: 40.15% vs. 16.59%) perform BSE and only 14.61% of respondents (urban vs. rural: 21.90% vs. 8.08%) had ever visited a screening facility center for CBE. Although more than 60% the respondents from urban and rural area believed that BSE is essential to detect breast cancer at early stage (Table 2). The bar diagram in Figures 2 and 3 shows that the frequency of checking breasts was very poor among the women. Around 77% (urban vs. rural: 65.94% vs. 86.46%) had never performed BSE and only 11.62% (urban vs. rural: 14.36% vs. 9.17%) checked their breasts monthly. More than 90% (urban vs. rural: 87.59% vs. 93.89%) not even visited health professionals for CBE.

**Table 2:** Breast cancer screening awareness and practice.

Variable	Urban (%)	Rural (%)	Total (%)
Have you ever heard about breast cancer?			
No	90 (21.90)	110 (24.02)	200 (23.01)
Yes	321 (78.10)	348 (75.98)	669 (76.99)
Total	411 (100%)	458 (100%)	869 (100%)
Is it a preventable disease?			
No	98 (23.84)	149 (32.53)	247 (28.42)
Yes	313 (76.16)	309 (67.47)	622 (71.58)
Total	411 (100%)	458 (100%)	86 9(100%)
Is it a severe disease?			
No	77 (18.73)	95 (20.74)	172 (19.80)
Yes	334 (81.27)	363 (79.26)	697 (80.20)
Total	411 (100%)	458 (100%)	869 (100%)
Have you ever heard about the breast clinical examination?			
No	149 (36.25)	264 (57.64)	413 (47.53)
Yes	262 (63.75)	194 (42.36)	456 (52.47)
Total	411 (100%)	458 (100%)	869 (100%)
Do you visit health professionals for breast clinical examination?			
No	321 (78.10)	421 (91.92)	742 (85.39)
Yes	90 (21.90)	37 (8.08)	127 (14.61)
Total	411 (100%)	458 (100%)	869 (100%)
**I**s breast self‐examination essential to detect breast cancer at an early age?			
No	46 (11.19)	52 (11.35)	98 (11.28)
Yes	287 (69.83)	238 (51.97)	525 (60.41)
Don't Know	78 (18.98)	168 (36.68)	246 (28.31)
Total	411 (100%)	458 (100%)	869 (100%)

### 3.3 Associated factors of breast self‐examination

In multivariate logistic regression, the higher the education level having among the women (AOR: 2.7 [95% CI: 1.6–4.6], AOR: 7.3 [95% CI: 3.9–13.4], and AOR: 8.1 [95% CI: 4.2–15.5] for secondary, higher secondary, higher education respectively) were more likely to perform BSE than having only primary education. Women having 20,001–40,000 family income were two times (AOR: 1.96 [95% CI: 1.2–3.0]) more likely to perform BSE than the lower income group (<20,000BDT). However, women residing in rural areas were approximately three times (AOR: 0.36 [95% CI: 0.25–0.52]) less likely to perform BSE than urban dwellers (Table 3).

**Table 3:** Factors associated with breast self‐examination.

Variables	BSE		Univariate		Multivariate	
	Performed, *n* (%)	Not performed, *n* (%)	OR (95% CI)	*p* Value	OR (95% CI)	*p* Value
Age group						
18–39 years	186 (77.18)	437 (69.59)	1		1	
40–49 years	37 (15.35)	89 (14.17)	0.97 (0.64–1.48)	0.913	1.48 (0.91–2.42)	0.113
50 & more years	18 (7.47)	102 (16.24)	0.41 (0.24–0.70)	0.001	0.80 (0.42–1.54)	0.522
Marital status						
Single	50 (20.75)	56 (8.92)	1	<0.001	1	0.648
Married	191 (79.25)	572 (91.08)	0.37 (0.24–0.56)		1.215974 (0.52–2.81)	
Types of family						
Nuclear	201 (83.40)	439 (69.90)	1	<0.001	1	0.054
Joint/extended	40 (16.60)	189 (30.10)	0.46 (0.31–0.67)		0.65 (0.42–1.00)	
Education status						
Primary	23 (9.54)	216 (34.39)	1		1	
Secondary	82 (34.02)	276 (43.95)	2.79017 (1.69–4.57)	<0.001	**2.734044** (1.60–4.67)	**<0.001** ***
Higher Secondary	60 (24.90)	72 (11.46)	7.82 (4.51–13.56)	<0.001	**7.31** (3.97–13.44)	**<0.001** ***
Undergraduate/Postgraduate	76 (31.54)	64 (10.19)	11.15 (6.47–19.20)	<0.001	**8.11** (4.25–15.50)	**<0.001** ***
Religion						
Muslim	228 (94.61)	586 (93.31)	1.25 (0.66–2.38)	0.484	**0.87** (0.42–1.78)	**0.706**
Hindu	13 (5.39)	42 (6.69)	1		1	
Family income						
<20,000BDT	41 (17.01)	201 (32.01)	1		1	
20,000‐40,000BDT	156 (64.73)	339 (53.98)	2.25 (1.53–3.31)	<0.001	**1.96** (1.26–3.03)	**0.003** **
40,001‐60,000BDT	23 (9.54)	58 (9.24)	1.94 (1.07–3.50)	0.027	0.96 (0.49–1.88)	0.912
More than 60,000BDT	21 (8.71)	30 (4.78)	3.43 (1.78–6.58)	<0.001	1.44 (0.67–3.08)	0.347
Occupational status						
Student	57 (23.65)	62 (9.87)	2.97 (1.99–4.43)	<0.001	1.64(0.73–3.70)	0.228
Housewife	172 (71.37)	557 (88.69)	1		1	
Private/public employee	12 (4.98)	9 (1.43)	4.31 (1.78–10.42	0.001	2.29 (0.83–6.32)	0.108
Living area						
Urban	165 (68.46)	246 (39.17)	1	<0.001	1	**<0.001** ***
Rural	76 (31.54)	382 (60.83)	0.29 (0.21–0.40)		**0.36** (0.25–0.52)	

*Note*: Bold values indicate statistically significant at *p* < 0.05.

Abbreviations: OR, odds ratio; 95% CI, 95% confidence interval.

** *p* < 0.05

*** *p* < 0.001.

### 3.4 Associated factors of clinical breast examination

Married women were more than three times (AOR: 3.2 [95% CI: 1.1–9.3]) more likely to perform CBE than single women. Women with higher secondary and higher education (AOR: 3.4 [95% CI: 1.8–6.6] and AOR: 3.0 [95% CI: 1.5–6.1], respectively) were more likely to perform CBE than those with primary education. However, women residing in rural areas were about three times (AOR: 0.37 [95% CI: 0.23–0.58]) less likely to perform CBE than urban dwellers (Table 4).

**Table 4:** Factors associated with clinical breast examination.

Variables	CBE		Univariate		Multivariate	
	Performed, *n* (%)	Not performed, *n* (%)	OR (95% CI)	*p* Value	OR (95% CI)	*p* Value
Age group						
18–39 years	100 (78.74)	523 (70.49)	1		1	
40–49 years	15 (11.81)	111 (14.96)	0.70 (0.39–1.26)	0.241	0.72 (0.39–1.34)	0.311
50 & more years	12 (9.45)	108 (14.56)	0.58 (0.30–1.09)	0.093	0.76 (0.37–1.58)	0.474
Marital status						
Single	15 (11.81)	91 (12.26)	1		1	
Married	112 (88.190	651 (87.74)	1.04 (0.58–1.86)	0.885	**3.25** (1.12–9.37)	**0.029** *
Types of family						
Nuclear	110 (86.61)	530 (71.43)	1		1	
Joint/extended	17 (13.39)	212 (28.57)	0.38 (0.22–0.65)	<0.001	0.57 (0.32– 1.02)	0.062
Education status						
Primary	22 (17.50)	217 (29.25)	1		1	
Secondary	39 (30.71)	319 (42.99)	1.20 (0.69–2.09)	0.505	1.25 (0.69–2.27)	0.452
Higher Secondary	33 (25.98)	99 (13.34)	3.28 (1.82–5.92)	<0.001	**3.44** (1.79–6.61)	**<0.001** ***
Undergraduate/Postgraduate	33 (25.98)	107 (14.42)	3.04 (1.69–5.47)	<0.001	3.02 (1.48–6.15)	0.002**
Religion						
Muslim	121 (95.28)	693 (93.40)	1.42(0.59‐3.40)	0.424	1.16 (0.46–2.92)	0.748
Hindu	6 (4.72)	49(6.60)	1		1	
Family income						
<20,000 BDT	32 (25.20)	21 (28.30)	1		1	
20,000–40,000 BDT	74 (58.27)	421 (56.74)	1.15 (0.73–1.80)	0.531	1.00 (0.61–1.63)	0.997
40,001–60,000 BDT	11 (8.66)	70(9.43)	1.03 (0.49‐2.15)	0.935	0.70 (0.31–1.56)	0.389
More than 60,000 BDT	10 (7.87)	41 (95053)	1.60 (0.73–0.50)	0.24	0.86 (0.35–2.09)	0.747
Occupational status						
Student	21 (16.56)	98 (13.21)	1.30 (0.77–2.18)	0.315	1.63 (0.62–4.28)	0.32
Housewife	103 (81.10)	626 (84.37)	1		1	
Private/public employee	3 (2.36)	18 (2.43)	1.01 (0.29–3.49)	0.984	0.56 (0.15–2.09)	0.391
Living area						
Urban	90 (70.87)	321 (43.26)	1		1	
Rural	37 (29.13)	421 (56.74)	0.31 (0.20– 0.47)	<0.001	**0.37** (0.23–0.58)	**<0.001** ***

*Note*: Bold values indicate statistically significant at *p* < 0.05.

Abbreviations: OR, odds ratio; 95% CI, 95% confidence interval.

* *p* < 0.05

** *p* < 0.01

*** *p* < 0.001.

### 3.5 Barriers toward breast cancer screening

**Figure 4:**
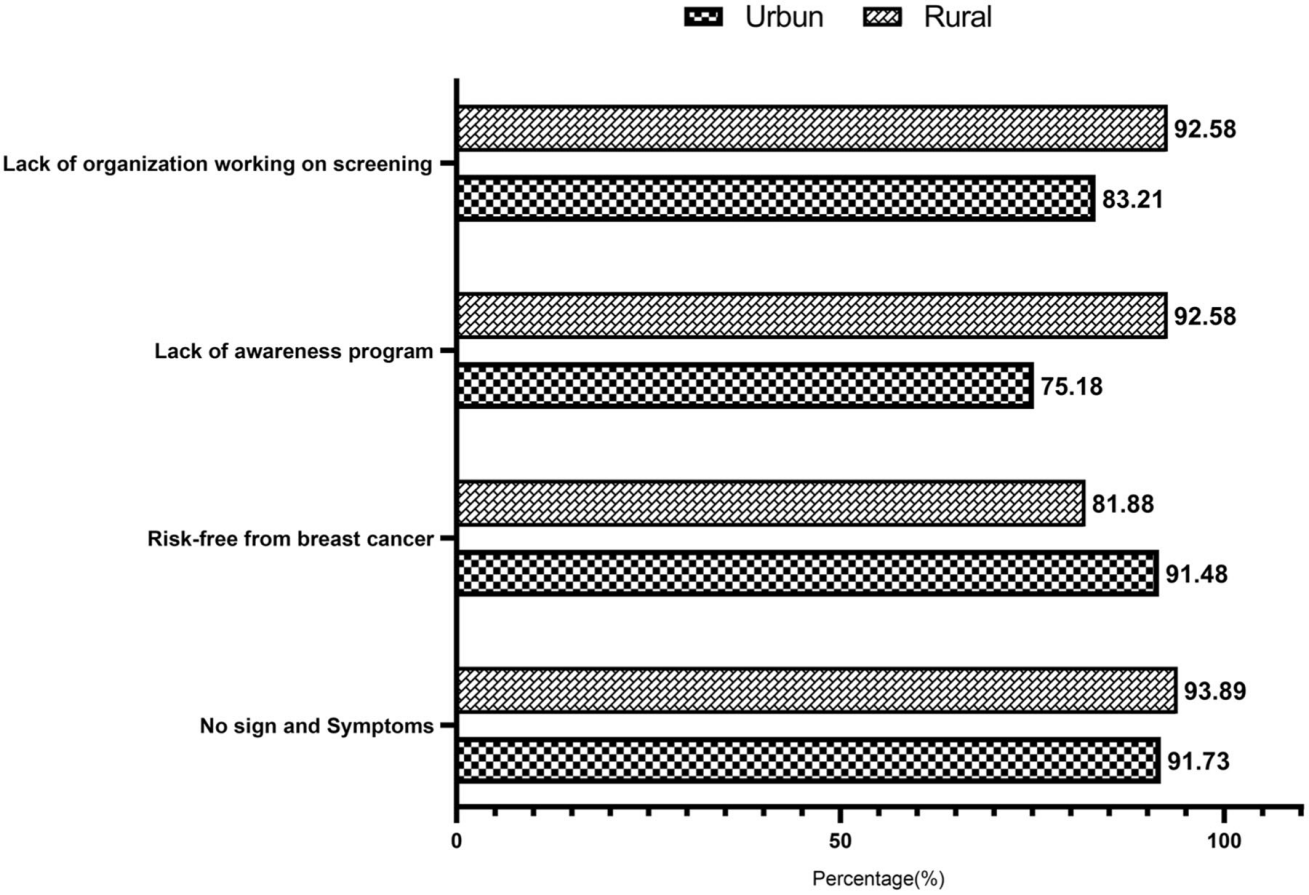
Perceived barrier toward breast cancer screening among the participants.

The bar diagram in Figure 4 shows the major perceived barriers toward breast cancer screening. Regarding the perceived barrier toward breast cancer screening, majority of the respondents did not feel any obstacles by “social stigma” (74.10%; urban vs. rural: 77.13% vs. 71.4%), “fear of clinical examination” (66.28%; urban vs. rural: 72.68% vs. 60.70%) and “shy to uncover their breasts” (66.97%; urban vs. rural: 71.95% vs 62.66%) for breast cancer screening. However, majority of the respondents felt “having no sign and symptoms” of breast cancer (92.86%; urban vs. rural: 91.73% vs. 93.89%) and “free of breast cancer risk” (86.42%; urban vs. rural: 91.48% vs. 81.88%) constrained them from screening. About 84.34% of respondents (urban vs. rural: 75.18% vs. 92.58%) reported “lack of awareness program” and 88.14% respondents (urban vs. rural: 83.21% vs. 92.58%) addressed “lack of governmental and nongovernmental organizations working on breast cancer screening” as the barriers to getting information on breast cancer screening.

## 4 DISCUSSION

This study is one of the few community‐based studies investigating awareness, practice, and perceived barriers to breast cancer screening among urban and rural women in Bangladesh. The study findings showed that despite having a high awareness of breast cancer, most women were unaware of breast cancer screening in terms of both BSE and CBE. The results agreed with the study conducted in Bangladesh and Ethiopia,^11^, ^21^ implying that awareness of screening for breast cancer needs to be addressed in Bangladesh.

The practice of BSE and CBE was even worse among the women in this region. A similar finding was found among university students in Bangladesh and in a Nigerian study.^14^, ^22^ However, a Malaysian study observed a different scenario where more than 50% of women practised both BSE and CBE.^23^ This gap in screening uptake in different regions probably awareness varieties on breast cancer screening among the respondents. A previous study showed educated and employed women were more aware of screening,^24^ as we found more women with higher education and higher income were living in the urban area with better screening awareness and practice than their counterparts. Raising awareness of the importance of screening, as shown by a recent study in Bangladesh, could be a key factor in increasing screening practice among women in this region.^9^ This study also reported the uptake of screening for breast cancer is poor in a rural area, which aligns with our findings that urban dwellers BSE and CBE practices proportion was almost three times better than rural dwellers. However, the breast cancer screening proportions were similar among urban and rural dwellers in developed countries like the United States.^25^ Current prediction analysis revealed the regional disparity. Rural dwellers were three times less likely to perform BSE and CBE than urban dwellers. Similar discrepancy was also found in BSE practice evident from recent studies from different parts of the world.^26^, ^27^, ^28^ On the contrary, a Vietnamese study reported no association was found between CBE uptake and area of residence.^29^ The possible explanation could be easy accessibility to information on screening and the tendency to seek more health care by the urban women than the rural women.

Our current study also explored other sociodemographic variables like education and family income. The women with secondary education and above were more likely to perform BSE and CBE than those with primary education. Evidence from other studies^11^, ^23^, ^30^ supported the significant association between educational attainment and better screening practice. In the case of the monthly income, women who have 20,001 to 40,000 BDT family income were two times (AOR: 1.96 [95% CI: 1.2–3.0]) more likely to perform BSE than the lower income group, but no association was found in CBE uptake which is consistent with other studies.^27^, ^29^ In contrast, married women were more prone to do breast cancer screening than the single women we found. A study reported marital status influenced the better utilization of breast, cervical, and colorectal screening.^31^ This is probably due to better breast cancer awareness among married women.^32^

Another aim of the study was to determine the perceived barrier toward screening along with the awareness and screening practice. The result of the study shows that most respondents did not feel obstructed in screening due to fear of clinical examination, social stigma, shyness of uncovering their breasts, and no time to do breast screening. However, a previous study in Bangladesh reported embarrassing to tell people and uncovering breasts were the leading barriers to screening.^12^ Some previous studies revealed that embarrassment, fear of screening, and lack of awareness were major impediments to breast cancer screening.^33^, ^34^ This dissimilarity of the findings could be due to differences in the study place. Our study confirmed that a lack of awareness programs was a major barrier to getting information about screening. Moreover, the perception of having no symptoms and being free from breast cancer risk discouraged them from going for screening, which is consistent with the findings from a previous large population‐based study in Bangladesh.^11^ This study findings could effectively design interventions on health education and health promotion programs for breast cancer screening in developing countries like Bangladesh.

One of the strengths of the study is using multistage sampling to select samples randomly from the population. A decent sample size was taken from urban and rural communities that may reflect the characteristics of the general population. Due to the cross‐sectional study design, clear evidence about causal association of risk factors with breast cancer screening cannot be ensured. Moreover, the self‐reported data were collected by an interviewer‐administered questionnaire. So, recall bias from respondents’ side and interviewer bias are possible.

## 5 CONCLUSION

This study found limited awareness and practice of breast cancer in both urban and rural dwellers women in Bangladesh. However, the awareness and practice were relatively higher among urban women. This study reveals educated women had better practising habits. Also, having no symptoms was a leading barrier to breast cancer screening, indicating poor awareness among the women. This study suggests implementing socially acceptable health education and health promotion interventions to extend awareness campaigns and scaling up the screening program at the community level.

## AUTHOR CONTRIBUTIONS

**Mohammad Injamul Hoq**: Conceptualization; methodology; writing—original draft; writing—review & editing. **Shamima Jahan**: Conceptualization; writing—original draft. **Md Hasan Mahmud**: Data curation; project administration; writing—original draft. **Md Mayin Uddin Hasan**: Data curation; formal analysis. **Md Jakaria**: Supervision; writing—review & editing.

## CONFLICT OF INTEREST STATEMENT

The authors declare no conflict of interest.

## ETHICS STATEMENT

Ethical approval was taken from the IRB of the University of Creative Technology Chittagong for this work (Ref: IRB/UCTC/2021/04).

## TRANSPARENCY STATEMENT

The lead author Mohammad Injamul Hoq affirms that this manuscript is an honest, accurate, and transparent account of the study being reported; that no important aspects of the study have been omitted; and that any discrepancies from the study as planned (and, if relevant, registered) have been explained.

## Data Availability

Data will be made available upon reasonable request.
